# Asymmetric bi-level dual-core mode converter for high-efficiency and polarization-insensitive O-band fiber-chip edge coupling: breaking the critical size limitation

**DOI:** 10.1515/nanoph-2024-0320

**Published:** 2024-09-09

**Authors:** Xiaolin Yi, Dongyue Sun, Weike Zhao, Hanwen Li, Long Zhang, Yaocheng Shi, Daoxin Dai

**Affiliations:** State Key Laboratory for Extreme Photonics and Instrumentation, College of Optical Science and Engineering, Center for Optical & Electromagnetic Research, International Research Center for Advanced Photonics, Zhejiang University, Zijingang Campus, Hangzhou 310058, China; Ningbo Research Institute, Zhejiang University, Ningbo 315100, China; Jiaxing Key Laboratory of Photonic Sensing & Intelligent Imaging, Intelligent Optics & Photonics Research Center, Jiaxing Research Institute, Zhejiang University, Jiaxing 314000, China

**Keywords:** edge coupler, asymmetric, dual-core, bi-level, silicon photonics, mode evanescent coupling

## Abstract

Efficient coupling between optical fibers and on-chip photonic waveguides has long been a crucial issue for photonic chips used in various applications. Edge couplers (ECs) based on an inverse taper have seen widespread utilization due to their intrinsic broadband operation. However, it still remains a big challenge to realize polarization-insensitive low-loss ECs working at the O-band (1,260–1,360 nm), mainly due to the strong polarization dependence of the mode coupling/conversion and the difficulty to fabricate the taper tip with an ultra-small feature size. In this paper, a high-efficiency and polarization-insensitive O-band EC is proposed and demonstrated with great advantages that is fully compatible with the current 130-nm-node fabrication processes. By introducing an asymmetric bi-level dual-core mode converter, the fundamental mode confined in the thick core is evanescently coupled to that in the thin core, which has an expanded mode size matched well with the fiber and works well for both TE/TM-polarizations. Particularly, no bi-level junction in the propagation direction is introduced between the thick and thin waveguide sections, thereby breaking the critical limitation of ultra-small feature sizes. The calculated coupling loss is 0.44–0.56/0.48–0.61 dB across the O-band, while achieving 1-dB bandwidths exceeding 340/230 nm for the TE/TM-polarization modes. For the fabricated ECs, the peak coupling loss is ∼0.82 dB with a polarization dependent loss of ∼0.31 dB at the O-band when coupled to a fiber with a mode field diameter of 4 μm. It is expected that this coupling scheme promisingly provides a general solution even for other material platforms, e.g., lithium niobate, silicon nitride and so on.

## 1 Introduction

Silicon photonics has gained substantial interest in recent years owing to its high integration density, cost-efficiency and complementary metal oxide-semiconductor (CMOS) compatibility [1]. This has made it an ideal solution for transmitting light in high performance optical interconnect systems. Within these complex optical transmission systems, the interfacing between photonic integrated chips and fibers is frequently used and highly desired, making it a crucial component of the whole system [2]. Nevertheless, the high-efficient coupling on the Silicon-on-insulator (SOI) platform still remains a big challenge, which is mainly due to the large mode mismatch between the sub-micron silicon waveguides and optical fibers.

To this end, considerable efforts have been made to realize efficient coupling by utilizing smart structural designs, including the evanescent coupler assisted with a special processed fiber [3], [4], polymer waveguide-based photonic wire bonding or 3D couplers [5], [6], [7], grating couplers [8], [9] and edge couplers (ECs) [10], [11], [12]. The first two categories typically offer high coupling efficiency, but are not compatible with the wafer-scale fabrication processes and increase the package difficulty. The approach of using grating couplers is an attractive option for large-scale integration and wafer-scale testing, yet it suffers from inherent narrow bandwidths, low coupling efficiency and polarization sensitivity. In contrast, ECs are increasingly important because of the capability with broad bandwidth, high efficiency and low polarization dependence, while additional edge dicing and polishing are required to achieve high-quality facets [13].

Currently attentions have been paid to develop various types of ECs for silicon photonics, including suspended cantilever structures [14], [15], SiN-assisted structures [16], [17], [18], multi-tip configurations [19], [20], subwavelength-grating (SWG) designs [21], [22], [23] and multi-stage tapers [24], [25], [26]. It can be seen that these reported structures are helpful to achieve high efficiency for the case of working at the C-band. However, for the popular O-band with shorter wavelengths around 1,310 nm than the C-band, it becomes even more challenging to develop high-efficiency ECs due to the following two reasons. First, smaller taper tips or a smaller pitch for the metamaterial waveguide at the chip facet are required to ensure a low-loss coupling because of the shorter wavelength. Second, realizing polarization-insensitive ECs remains difficult even with a small tip width, because the TM polarization mode has tighter confinement than the TE polarization mode in a narrow waveguide tip.

Recently, some efforts have been made to address these challenges. A suspended metamaterial-waveguide-based EC for the O-band is proposed with a minimal feature size of 120 nm, showing a peak coupling loss of 1.3 dB with polarization dependent loss (PDL) of 0.3 dB [27]. However, this is designed with a 150-nm-thick silicon core layer, in which case an additional transition is required to connect the popular 220-nm-thick SOI photonic waveguide for practical applications. Alternatively, a silicon inverse taper and a low-index SiON ridge waveguide are combined to efficiently expand the mode field. The silicon tip width at the interface of the silicon waveguide and the SiON waveguide is 130 nm, achieving a coupling loss of ∼4 dB and PDL of ∼2 dB when coupling to an SMF [28]. Another suspended EC was demonstrated by using a parabolic inverse taper with an ultra-small tip width of only 70 nm, showing a coupling loss of 2–3 dB for TE polarization mode only at 1,280–1,340 nm [29]. A dual-tip SiN EC combined with a SiN–Si transition structure is also introduced to realize the coupling between a 4-μm mode field diameter (MFD) fiber and a silicon photonic waveguide [30], where the silicon tip width at the SiN–Si transition interface is about 120 nm, achieving an overall coupling loss of 2.35 dB and 3–4.8 dB for TE and TM polarization modes, respectively. Notably, despite the potential of the bi-level inverse taper structure for achieving low-loss edge coupling [31], [32], the key challenge is creating a very tiny tip at the bi-level junction between the thick and thin silicon core regions to improve the coupling efficiency for the TM polarization mode (see the simulation result in Supplementary S1), which imposes critical fabrication requirements. On the other hand, unfortunately, even with an ultra-small waveguide tip size, the improvement in the coupling efficiency for the TM polarization mode is still limited. For example, there is a mode conversion loss of ∼0.2 dB from the thick core to the thin core even when the tip size is reduced to only 40 nm. As a summary, it is still highly desired to achieve a polarization-insensitive low-loss edge coupling for silicon photonic chips working at the O-band with relaxed requirements for the fabrication.

In this paper, we propose a high-efficiency and polarization-insensitive O-band EC on the standard 220-nm SOI platform by introducing an asymmetric bi-level dual-core mode converter, which is fully compatible with the current regular 130-nm fabrication node. The introduced asymmetric bi-level dual-core mode converter enables low-loss mode expansion for both TE and TM polarization modes confined in the thick-core silicon photonic waveguide to be matched well with the fiber and works well for both polarizations. Particularly, there is no bi-level junction in the propagation direction introduced between the thick and thin sections of the inverse taper, thereby breaking the critical limitation for the small feature size required in the previous bi-level inverse tapers [31], [32]. The calculated coupling loss to a high numerical aperture (HNA) fiber with an MFD of ∼4 μm are 0.44–0.56 dB and 0.48–0.61 dB for TE and TM polarization modes across the O-band, respectively, while their 1-dB bandwidths are even more than 340 nm and 230 nm. For the fabricated ECs, the experimental results show a peak coupling efficiency of ∼0.82 dB with a PDL of ∼0.31 dB at the O-band, thus providing a general solution for the fiber-chip coupling with easy fabrication.

## 2 Principle and design

**Figure 1:**
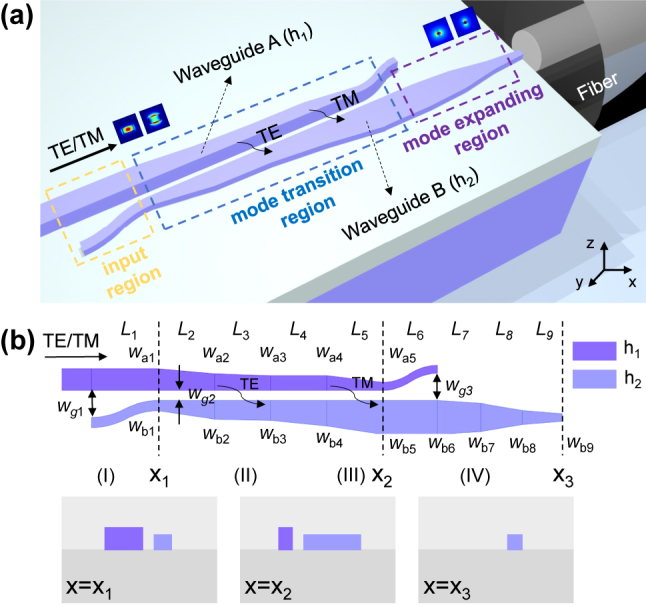
Illustration of the proposed EC based on an asymmetric bi-level dual-core mode converter. (a) 3D illustration. (b) The top view of the EC and the cross sections of the silicon photonic waveguides at different sections.

Figure 1(a) shows the 3D illustration of the proposed asymmetric bi-level dual-core mode converter for efficient coupling, which consists of two adjacent cores with different heights (*h*_1_, *h*_2_; *h*_1_ > *h*_2_). Note that the mode converter includes nine sections, whose parameters are shown in Figure 1(b), including the input region (Section 1), the mode transition region (Sections 2 and 3) and the mode expanding region (Section 4). At Section 1 for the input region, the fundamental TE and TM modes are launched from the thick waveguide (A) whose height is *h*_1_. Then they are efficiently coupled to the thin waveguide (B) whose height is *h*_2_ through Section 2 for the TE polarization mode coupling and Section 3 for TM polarization mode coupling, respectively. Finally, the mode field is expanded at the mode expanding Section 4. In this way, both TE and TM polarization modes can be converted efficiently from the thick singlemode waveguide to the thin narrow tip, which has an expanded mode size matched well with the fiber. It is worth noting that both the bi-level inverse taper structure and the proposed coupling structure require an additional overlay etch step to form the thin core. Such an etching step is compatible with the standard CMOS fabrication process, which is similar to that used frequently for fabricating grating couplers. When the silicon core is thin, a relatively large tip width is allowed for high mode coupling efficiency (see Supplementary S1). The fabrication for the structures with larger feature sizes becomes easier, particularly for MPW processes. Besides, it is possible to enhance the overlay tolerance further by choosing a larger gap width when needed. In particular, no bi-level junction in the propagation direction is introduced between the thick and thin sections of the inverse taper, avoiding to introduce any ultra-tiny tip, and thus it is fully compatible with the current 130-nm fabrication node and shows great potential for large-scale photonic systems on-a-chip.

Here the present EC is designed on the standard SOI platform with a 220-nm-thick top-silicon layer, which is one of the most popular options for silicon photonics. The refractive index of the silicon core and the SiO_2_ cladding are *n*_si_ = 3.506 and *n*_sio2_ = 1.446 at around 1,310 nm, respectively. Accordingly, the height *h*_1_ of the thick waveguide A is chosen as 220 nm. The width *w*_a1_ of the input singlemode waveguide is chosen as 0.38 μm. The width *w*_b1_ of the input S-bend waveguide should be small enough so that the mode is mainly localized in the thick waveguide A, which is chosen as 0.18 μm here for fabrication ease. The gap *w*_g2_ between two waveguides is also chosen as 0.18 μm for fabrication ease. The gap *w*_g1_ is chosen as 2 μm, which is sufficiently large to avoid coupling from the other waveguide. The length *L*_1_ of the input Section 1 is chosen as 10 μm, which is sufficiently long for the S-bend to have adiabatic low-loss transmission.

The overall coupling loss can be divided into two parts, including the mode mismatch loss *α*_c_ between the mode at the chip facet and the optical fiber, and the on-chip mode transmission loss *α*_t_ from the singlemode waveguide to the chip facet. These two parts are carefully optimized, as described below.

### 2.1 Mode mismatch loss

The mode coupling efficiency *η* and the mode mismatch loss *α*_c_ between the modes at the chip facet and the optical fiber by using the following formula:

(1) $$\begin{array}{cc} \eta & =\frac{|\int E_{1}E_{2}\text{d}A{|}^{2}}{\int |E_{1}{|}^{2}dA\int |E_{2}{|}^{2}dA}\\ \alpha_{\text{c}} & =10Log_{10}\eta , \end{array}$$

where *E*_1_ and *E*_2_ is the complex electric distributions at the chip facet and the fiber, respectively. We calculate the mode mismatch loss *α*_c_ and PDL at the wavelength of 1,260, 1,310 and 1,360 nm by varying the tip width *w*_b9_ and the core height *h*_2_ of the thin waveguide B, respectively. The height *h*_2_ of the thin waveguide B is chosen as 150 nm, which is also compatible with the standard MPW fabrication process of many foundries. The tip width *w*_b9_ is then optimally chosen as 130 nm, and the mode mismatch loss *α*_c_ is as low as 0.22/0.20 dB for TE/TM polarization mode, respectively. More details are given in Supplementary S1.

### 2.2 On-chip mode transmission loss

The total on-chip mode transmission loss *α*_t_ includes the transmission loss in the mode transition region (*α*_tt_) and the mode expanding region (*α*_te_). For the mode transition region, the widths *w*_ai_ and *w*_bi_ (*i* = 2, 3, 4, 5) of the cores in Sections 2 and 3 should be designed carefully, so that the TE and TM polarization modes can be evanescent coupled to the thin waveguide B with low loss. For the TE polarization mode coupling Section 2, the core width should be chosen optimally to make *n*_eff_ (*w*_a2_) > *n*_eff_ (*w*_b2_) and *n*_eff_ (*w*_a3_) < *n*_eff_ (*w*_b3_) satisfied simultaneously. Similarly, for the TM-polarization mode-coupling Section 3, one should have *n*_eff_ (*w*_a4_) > *n*_eff_ (*w*_b4_) and *n*_eff_ (*w*_a5_) < *n*_eff_ (*w*_b5_) as well. The lengths of all the mode transition regions are optimized carefully to be adiabatic. We perform a comprehensive simulation of the entire mode transition region by using the finite-difference time-domain (3D-FDTD) method, see Supplementary S2. The simulation of the conventional bi-level inverse taper structure is also carried out as a comparison, see Supplementary S3. For the TE polarization mode, the transmission loss *α*_tt_ is as low as <0.045 dB in an ultra-broad bandwidth of over 300 nm. The reflection of the bi-level inverse taper is about −32.2–48.3 dB when the critical dimension *w*_min_ varies from 40 nm to 180 nm, which is around 10 dB higher than that observed in our proposed coupling scheme (<−57.1 dB) with an asymmetric bi-level dual-core mode converter. For the TM polarization mode, the transmission loss *α*_tt_ is <0.057 dB and the reflection is <−57.1 dB in the O-band operation when the tip width *w*_min_ is no more than 140 nm. As it should be noticed, the transmission loss of the conventional bi-level inverse taper even with **40**-nm tip size is about 0.08–0.17 dB, which is still higher than that (∼0.01–0.05 dB) of our present asymmetric bi-level dual-core mode converter with **130**-nm tip size. The simulation results reveal that the present EC performs much better than the conventional bi-level inverse taper when the tip width is chosen to be more than 130 nm (which is a typical fabrication node of foundries). For example, the loss of the present mode converter remains at a low level of <0.24 dB even when *w*_min_ is increased to 160 nm, while the conventional bi-level inverse taper has a loss as high as 0.83–0.91 dB. More details are given in Supplementary S2 and S3. Additionally, the fabrication tolerance analysis of the mode transition region shows that the maximum transmission loss of 0.25 dB is observed when the width variation Δ*w* = +20 nm, see Supplementary S4.

For the mode expanding region (Section 4), it is designed by being divided into several inverse-taper sections so that the footprint can be minimized. All the taper lengths (*L*_6_, *L*_7_, *L*_8_, *L*_9_) for these sections are carefully chosen to minimize the on-chip transmission loss *α*_te_. For the section at the facet, there is a trade-off between the mode conversion loss and the substrate leakage loss as the mode field is mainly expanded to be beyond the confinement of the core region, and thus the length *L*_9_ should be determined carefully. We give an analysis on the influence of the length *L*_9_ and the buried oxide layer thickness *h*_BOX_, and it can be seen that the substrate leakage loss is reduced to 0.08–0.24 dB when *L*_9_ is chosen as 50 μm considering *h*_BOX_ = 3 μm, while the transmission loss *α*_te_ is as low as 0.26/0.27 dB for TE/TM polarization mode. It is crucial to mention that the substrate leakage still increases slightly, especially at longer wavelengths, as the length *L*_9_ increases. Even though increasing the BOX thickness further, for example, to 4 μm, helps reduce the substrate leakage, we have considered a design with *h*_BOX_ = 3 μm, which is a commonly available commercial value. More details are given in Supplementary S5.

### 2.3 Overall coupling loss

**Figure 2:**
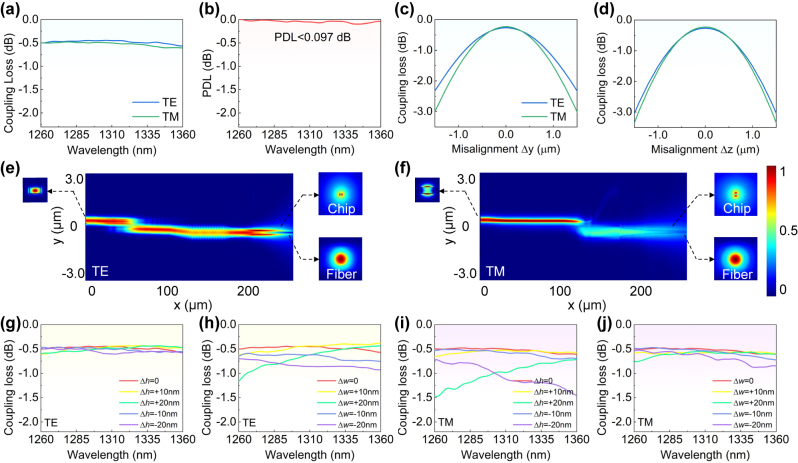
Analysis of the overall coupling loss. (a) Simulated overall coupling loss for TE/TM polarization mode. (b) Calculated PDL. Analysis of the misalignment tolerance for the present EC in the horizontal (c) and vertical (d) directions. (e) and (f) Simulated light propagation for TE/TM polarization mode. (g)–(j) Fabrication tolerance analysis when assuming that the core height or width has a deviation of ±10 nm and ±20 nm.

Finally, the overall coupling loss (which is the sum of the mode mismatch loss *α*_c_ and on-chip mode transmission loss *α*_t_) and the PDL are calculated, as shown in Figure 2(a) and (b). The simulated coupling loss is 0.44–0.56 dB and 0.48–0.61 dB in the wavelength range of 1,260–1,360 nm for TE and TM polarization modes, respectively. A low PDL of <0.097 dB is achieved across the O-band. Here the simulation is carried out under an ideal condition where the fiber is in close contact with the chip. While, it is more likely to have a small gap between them in reality. When considering a gap of 0.5 μm, an additional loss of 0.40 dB and 0.36 dB for TE and TM polarization mode is introduced. More details can be found in Supplementary S6. Figure 2(c) and (d) show the analysis of the misalignment tolerance to the center of the optical fiber, indicating that the 1-dB alignment tolerances in the horizontal and vertical directions are ∼±1.0 μm and ±0.9 μm, respectively. The simulated light propagations for TE/TM polarization modes are shown in Figure 2(e) and (f). As it can be seen, when TE/TM polarization modes are launched at the thick waveguide A, the evanescent coupling to the thin waveguide B occurs at Sections 2 and 3, significantly reducing the mode confinement in the vertical direction. The mode field undergoes further expansion through the inverse taper Section 4, allowing it to be matched well with the optical fiber.

The fabrication tolerance analysis is also carried out by assuming that the core height or width has a deviation of ±10 nm and ±20 nm, respectively, and the simulated results are shown in Figure 2(g–j). It can be seen that a loss increase is observed at short wavelengths for both TE and TM polarization modes when core-width deviation Δ*w* = +20 nm, because the mode confinement becomes stronger and the mode expansion becomes less. When Δ*w* = −20 nm, the loss deterioration of TE polarization mode mainly comes from the increased mode mismatch loss (∼0.74 dB), while the transmission loss *α*_tt_ remains at a low value of ∼0.01 dB. For the TM polarization mode, higher substrate leakage loss of ∼0.30 dB and higher mode mismatch loss *α*_c_ of ∼0.32 dB both contribute to the higher coupling loss. Note that the TM polarization mode coupling loss increases at the longer wavelength when the core-height deviation Δ*h* < 0, as indicated by the purple line. Here the coupling loss at 1,360 nm reaches 1.46 dB, which comprises of 0.45 dB mode mismatch loss *α*_c_, 0.06 dB on-chip transmission loss *α*_tt_ of the mode transition region and 0.95 dB transmission loss *α*_te_ of the mode expanding region, as given in Supplementary S2 and S3. The increased loss at the shorter wavelength when Δ*h* = +20 nm mainly owes to the increased mode mismatch loss, as shown by the green line. It can be seen that the TE polarization mode has relatively high sensitivity to the width deviation, while the TM polarization mode is predominantly affected by the core-height deviation. In addition, we also calculate the coupling loss in a broad wavelength-band ranging from 1,260 nm to 1,600 nm, demonstrating that the 1-dB bandwidth is as large as ∼340/230 nm for TE/TM polarization modes. More details can be found in Supplementary S7.

## 3 Fabrication and measurement

**Figure 3:**
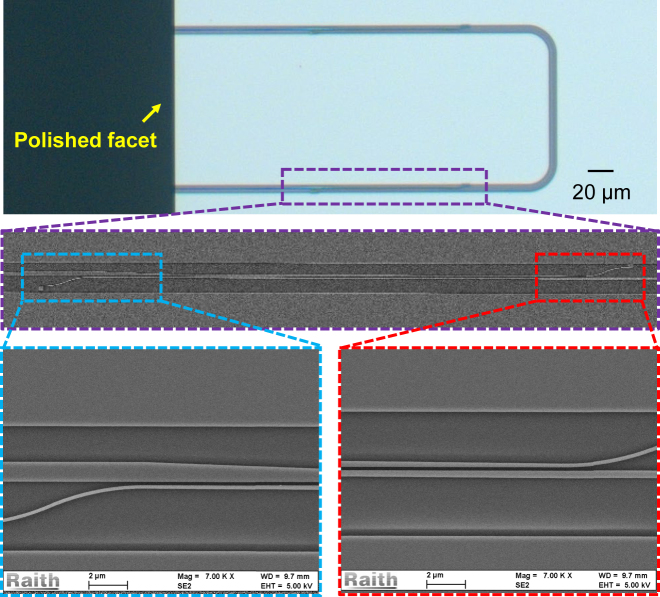
Microscope image and the SEM image of the fabricated EC.

The designed EC was fabricated on the SOI platform with a 220-nm-thick top-silicon layer and a 3-μm-thick buried oxide layer. The electron beam lithography (EBL) was used for the patterning and the inductively coupled plasma (ICP) dry-etching process was used for etching the silicon core. A 3-μm-thick SiO_2_ thin film was deposited above the Si layer as the cladding by utilizing the plasma enhanced chemical vapor deposition (PECVD) process. Figure 3 shows the microscope image and the scanning electron microscope (SEM) image of the fabricated EC with loop-back structure. The lateral separation between these two identical ECs is 127 μm.

**Figure 4:**
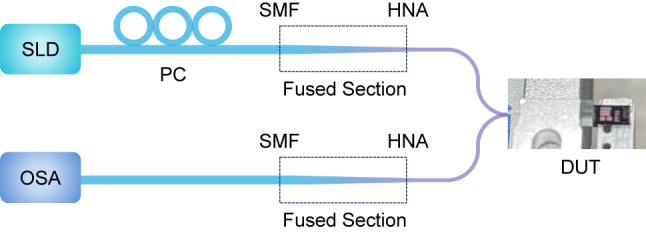
Experiment setup for the characterization of the EC. SLD, super luminescent diode; PC, polarization controller; HNA, high numerical aperture fiber; DUT, device under test; OSA, optical spectrum analyzer.

Figure 4 shows the experiment setup for the characterization of the EC. A Super Luminescent Diode (SLD) was used as the light source whose wavelength ranges from 1,260 nm to 1,360 nm. The polarization of the input light is adjusted by a polarization controller (PC). The TE- and TM-polarizations are established by adjusting the PC, and the polarizations are determined by using a polarimeter (PAX1000IR2) at the output port and analyzing the recorded power simultaneously. A 127 μm pitched HNA fiber array fused with SMF-28 was used to couple light in and out of the chip, which matches the distance between the two identical ECs. The total excess loss of the FA is ∼0.4 dB/channel (including the fusion splicing loss) and is subtracted from the coupling loss. The transmission spectrums are recorded by the optical spectrum analyzer (OSA).

**Figure 5:**
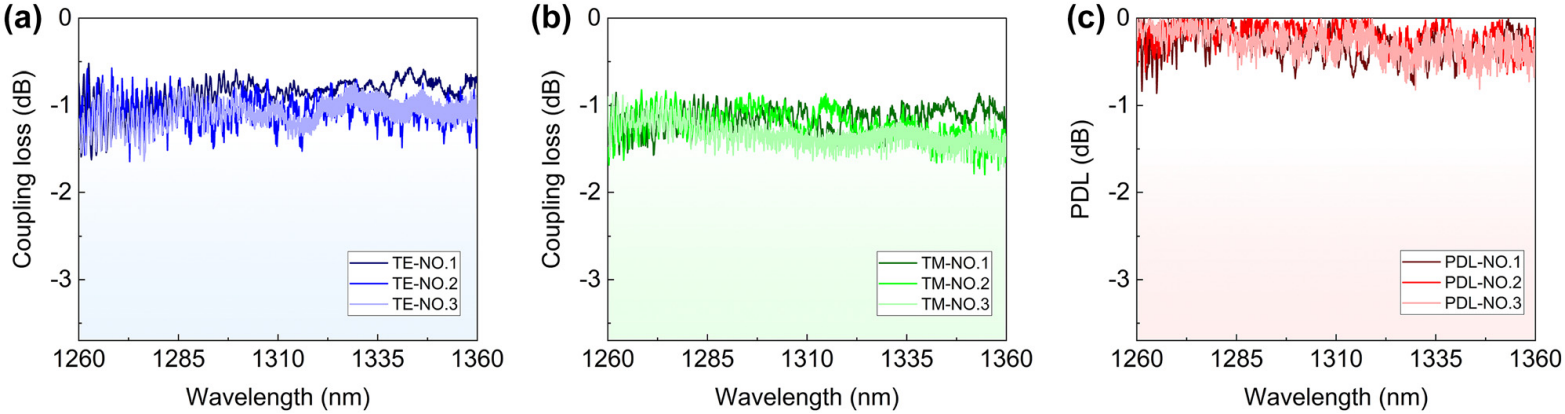
Measured coupling loss for TE (a) and TM (b) polarization mode and PDL (c) of the fabricated EC.

Figure 5(a–c) show the measured coupling loss and the calculated PDL of the fabricated EC. We measured three EC test structures, which are shown in different colors. The measured coupling loss is about 0.82–1.35 dB and 0.94–1.49 dB for TE- and TM-polarization modes while the PDL is ∼0.31 dB in the wavelength range of 1,260–1,360 nm. Note that there is some deviation from the simulated results, about 0.6 dB of loss deterioration is observed. On one hand, this may attribute to the fabrication imperfectness, while it is possibly because the core height and width has a deviation of 10–20 nm, which contributes to ∼0.2–0.4 dB additional loss, as analyzed in the fabrication tolerance result in Figure 2. On the other hand, the imperfect characterization process may also account for part of the loss increase. As it is well known, if the angle of the fiber array is not adjusted properly, it may also have influence on the misalignment on the *z* direction. For example, an angle deviation of ∼0.2° corresponds to a misalignment of ∼0.5 μm, leading to an additional loss of ∼0.3 dB, as shown in Figure 2. Besides, it is likely to have a ∼0.5 μm air-gap between the fiber and the chip during the characterization, which may result in 0.31–0.43 dB loss degradation, as discussed in Supplementary S6. The observed ripples at the longer wavelengths should be attributed to the end-to-end facet Fabry–Perot resonances caused by air-gap reflection. The ripples at the shorter wavelengths (∼1,260–1,300 nm) are possibly from the SLD source used in the experiment.

## 4 Discussion

Generally, the coupling loss, polarization dependence, operating bandwidth, device footprint, fabrication tolerance and misalignment tolerance are often used for evaluating the performance of the ECs. One should be aware that the fabrication ease is extremely important for the photonic devices/circuits developed for real applications. For example, those ECs that utilizing 3D polymer waveguide assisted structures, photonic wire bonding or special possessed fibers [4], [6], [7] still remains challenges for practical uses in view of the current fabrication capability available for the MPW foundries. While, those ECs that exhibit CMOS compatibility such as suspended waveguides, SiN-assisted structures or bi-level inverse tapers [29], [30], [32], usually requires a small feature size or additional complicated processes. It becomes even more challenging for operating at the short wavelength with dual polarizations, e.g. the O-band as discussed, because very tiny tip size is needed.

Table 1 gives a comparison between this work and the O-band silicon ECs reported previously. Our proposed EC utilizing the asymmetric bi-level dual-core mode converter shows low coupling loss and low polarization dependence in both simulation and experiments, compared with the other coupling structures. This is very important for photonic chips developed to work polarization-insensitively or to work with dual polarizations, which is often desired in data transmissions. Notably, the present asymmetric bi-level dual-core mode converter offers prospective broadband operation over 300 nm, potentially paving the way for ultra-broadband coupling in a wide range of applications, e.g., on-chip spectrometers [33] and optical frequency comb generators [34]. It can be seen that the present EC works well across both polarizations without additional undercut processes or introducing special low-index waveguides, while only a standard two-step etching process is required. This contributes to a high reliability for the photonic packaging, yielding high consistency and robustness in the performance of the optical system. More importantly, it should be pointed out that the minimum feature size is as large as 130 nm, which is fully compatible with the current 130-nm fabrication node. The coupling loss is also insensitive to this critical size across a broad range of width deviation, showcasing large fabrication tolerances.

**Table 1:** Comparison of the O-band silicon EC.

Ref.	Structure	Fiber	MFD	Coupling loss (dB)		PDL (dB)		Bandwidth (nm)		Minimum feature	Fabrication
		type	(μm)	Sim.	Exp.	Sim.	Exp.	Sim.	Exp.	size (μm)	
[27]	SWG (150 nm-thick)	SMF	10	–	1.3–2.1	–	0.5	–	100	0.12	Under cut
[28]	SiON lens	SMF	10	–	4.5–5.5	–	1–2	–	220	0.13	SiON assisted
[29]	Inversed taper	SMF	10	0.5–0.6	1.6–3.5	–	–	60	60	0.07	Under cut
[30]	Multicore-SiN	HNA	4	0.8–1.0	2.35	0.2–0.5	0.6–2.5	100	100	0.12	SiN assisted
**This**	**Asymmetric bi-level dual-core**	**HNA**	**4**	**0.44–0.56**	**0.82–1.35**	**<0.096**	**0.31**	**340/230**	**100**	**0.13**	**Two-step etching**

Further improvement can be made on the device footprint and the misalignment tolerance. The length of the present EC reaches 270 μm, which can be downsized by further optimizing the parameters of each section carefully. Achieving high-efficiency ECs with compact footprints is the key to overcome the photonic packaging bottleneck impeding by high-cost and limited throughput [2]. The misalignment tolerance can be improved if combined with the multi-core structures at the mode expanding region, which has been used for SWG-assisted ECs [20]. The idea of utilizing mode evanescent coupling can also be employed in combination with SiN-assisted structures, facilitating polarization-insensitive coupling in a broad bandwidth. It also has the potential for on-demand coupling with SMFs or fibers with different MFDs, where the predominant limitation lies on the non-negligible substrate leakage loss, attributing to the limited buffer layer thickness. Evidently, the idea of utilizing mode evanescent coupling all-on-chip provides a general solution even for other material platforms and other coupling cases, e.g., laser-chip coupling and chip-chip coupling on silicon nitride [35], lithium niobate [36], lithium tantalate [37] and so on. Particularly, even for optical waveguides with angled sidewalls (e.g., thin-film lithium niobate optical waveguides), the structure can also work well when the gap width is chosen to be relatively large. The remarkable fiber-chip coupling ability and the fabrication compatibility of the proposed EC makes it a promising candidate in next-generation silicon photonic circuits where power consumption matters mostly. We believe that this coupling approach opens the door for further implementations at the high-volume manufacturing and large-scale integration in the future.

## 5 Conclusions

In summary, we have proposed and demonstrated a low-loss and polarization-insensitive O-band fiber-chip EC that breaks the limitations on critical requirements for tiny feature size. The asymmetric bi-level dual-core mode converter enables low-loss mode conversion between the thick and thin silicon photonic waveguides. In particular, it avoids the introduction of bi-level junction, thereby reducing the fabrication difficulty. The calculated coupling loss is 0.44–0.56 dB and 0.48–0.61 dB across the O-band for TE and TM polarization modes, while their 1-dB bandwidths exceed 340 nm and 230 nm, respectively. The experimental results have shown that a peaking coupling loss is achieved with ∼0.82 dB and the PDL is about 0.31 dB in the O-band. This high-efficiency and polarization-insensitive EC has the potential for not only fiber-chip coupling but also various kinds of optical interface cases.

## Supplementary document

See Supplementary 1 for supporting content.

## Supplementary Material

Supplementary Material Details
