# Supplementary material for: Asymmetric bi-level dual-core mode converter for high-efficiency and polarization-insensitive O-band fiber-chip edge coupling: breaking the critical size limitation
Source: Nanophotonics. 2024 Sep 9;13(22):4149–57. doi: 10.1515/nanoph-2024-0320 (PMC11501050; doi:10.1515/nanoph-2024-0320)
Supplement: Supplementary file 1 — Supplementary Material Details [file j_nanoph-2024-0320_suppl_001.pdf]

Supporting Information

Asymmetric bi-level dual-core mode converter for high-efficiency and polarization-insensitive O-band fiber-chip edge coupling: breaking the critical size limitation

Xiaolin Yi[1], Dongyue Sun[1], Weike Zhao,[1] Hanwen Li[1], Long Zhang[1], Yaocheng Shi[1,2] and Daoxin Dai[1,2,3*]

[1]*State Key Laboratory for Extreme Photonics and Instrumentation, College of Optical Science and Engineering, Center for Optical & Electromagnetic Research, International Research Center for Advanced Photonics, Zhejiang University, Zijingang Campus, Hangzhou 310058, China*

[2]*Ningbo Research Institute, Zhejiang University, Ningbo 315100, China.*

[3]*Jiaxing Key Laboratory of Photonic Sensing & Intelligent Imaging, Intelligent Optics & Photonics Research Center, Jiaxing Research Institute, Zhejiang University, Jiaxing 314000, China*

*dxdai@zju.edu.cn

S1. Calculated mode mismatch loss at the wavelength of 1260, 1310 and 1360 nm

Figure S1(a-i) show the calculated mode mismatch loss α_c and polarization dependent loss (PDL) at the wavelength of 1260, 1310 and 1360 nm by varying the tip width w_{b9} and the core height h_2 of the thin waveguide B. It can be seen that, when considering the popular case with a core height of 220 nm, the optimized tip widths for TE and TM polarization modes are 0.11 μm and <0.06 μm, indicating that the scheme with a regular inverse taper cannot realize low coupling loss and low PDL for both polarizations simultaneously. In contrast, when the silicon core is thin, a relatively large tip width is allowed for high mode coupling efficiency. Unfortunately, one should be aware that the substrate leakage loss becomes higher as the core gets thinner, regarding that the buffer layer thickness is usually 2-3 μm. As a trade-off, here the height h_2 of the thin waveguide B is chosen as 150 nm, which is also compatible with the standard MPW fabrication process of many foundries. The tip width w_{b9} is then optimally chosen as 130 nm, and the mode mismatch loss α_c is as low as 0.22/0.20 dB for TE/TM polarization mode at the wavelength of 1310 nm, respectively. Besides, the TM polarization mode is less sensitive to the width deviation than the TE polarization mode because of their different mode profiles due to the boundary conditions. The 1-dB fabrication tolerance for the core width/height variation is as large as ±30/±60 nm and ±60/±30 nm for TE and TM polarization modes, respectively. The mode mismatch losses α_c at the wavelengths of 1260 nm and 1360 nm are calculated as ~0.19/0.31 dB and ~0.35/0.24 dB for TE/TM polarization modes, respectively.

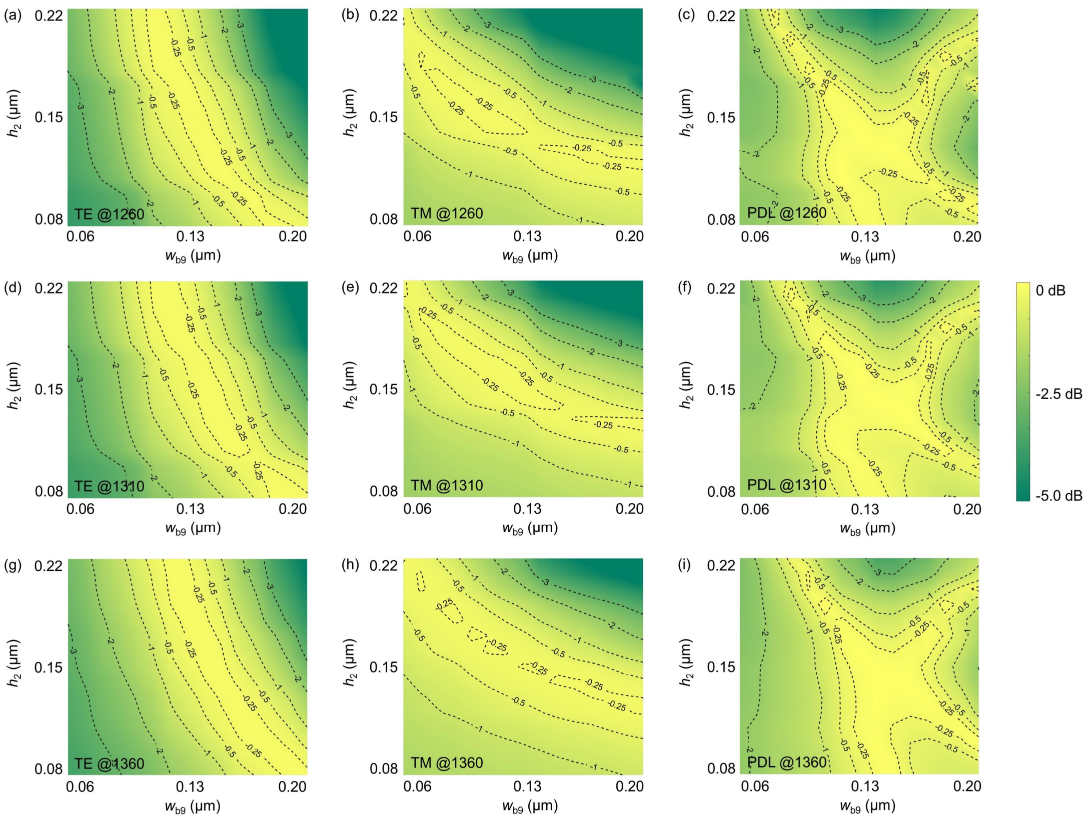

Figure S1: Calculated mode mismatch loss α_c and PDL at the wavelength of (a)-(c) 1260 nm, (d)-(f) 1310 nm and (g)-(i) 1360 nm.

S2. Simulation of the mode transition region of the asymmetric bi-level dual-core mode converter

Figure S2(a) and (b) show the calculated effective indices of waveguide A and B as the core width varies when operating at the wavelength of 1310 nm. Accordingly, the widths (w_{a2}, w_{a3}, w_{b2}, w_{b3}) are optimally chosen as (0.38, 0.26, 0.33, 0.35) μm. Notably, the effective index for TM polarization mode increases more slowly for the case with a lower height as the core width increases (see the green dashed line), indicating that a larger core width w_{b5} and a smaller core width w_{a5} should be chosen to satisfy the requirement. In this case, these widths (w_{a4}, w_{a5}, w_{b4}, w_{b5}) are optimally chosen as (0.26, 0.13, 0.45, 0.80) μm.

The lengths of all the mode transition regions are optimized carefully to be adiabatic. Here an eigenmode expansion (EME) solver is used to simulate the transmission. The length L_2 of the taper section is chosen as 20 μm to facilitate the adiabatic conversion from the input section to the TE coupling region. The length L_4 of the section connecting the TE and TM mode coupling regions is chosen optimally as 40 μm for low-loss adiabatic transmission as well. For the TE and TM mode coupling regions, the lengths L_3 and L_5 are optimized carefully to have sufficient evanescnet coupling from the thick waveguide A to the thin waveguide B. Figure S2(c) and (d) show the calculated transmission loss at different wavelengths in the TE/TM mode coupling sections II/III as the length L_3/L_5 varies. The lengths L_3 and L_5 is optimally chosen as 40 μm and 30 μm to realize adiabatic evanescent coupling.

We perform a comprehensive simulation of the entire mode transition region, and the simulated light propagation in sections II and III are shown in Figure S2(e) and (f), respectively. A finite-difference time-domain (3D-FDTD) method was used here for the calculation. Evidently, the launched mode

experiences a successful conversion from the thick waveguide A (h=220 nm) to the thin waveguide B (h=150 nm) for both polarizations. The transmission loss α_{tt} and the reflection are also depicted in Figure S2(g-j). Here the operation wavelength is varied from 1260 nm to 1600 nm, and the width w_{min} is the critical dimension in the device, i.e., w_{a5} in section III. As shown in Figure S2(g) and (h), for the TE polarization mode, the transmission loss α_{tt} is as low as <0.045 dB in an ultra-broad bandwidth of over 300 nm, while the reflection caused by the tip is about −45.6-−54.9 dB across the entire wavelength band of 1260-1600 nm even when the tip width w_{min} is increased to 180 nm. For the TM polarization mode, as shown in Figure S2(i) and (j), the transmission loss α_{tt} is <0.057 dB and the reflection is <–57.1 dB in the O-band operation when the tip width w_{min} is no more than 140 nm. When operating at the longer wavelength, the transmission loss slightly increases to 0.2-0.3 dB due to the weaker mode confinement.

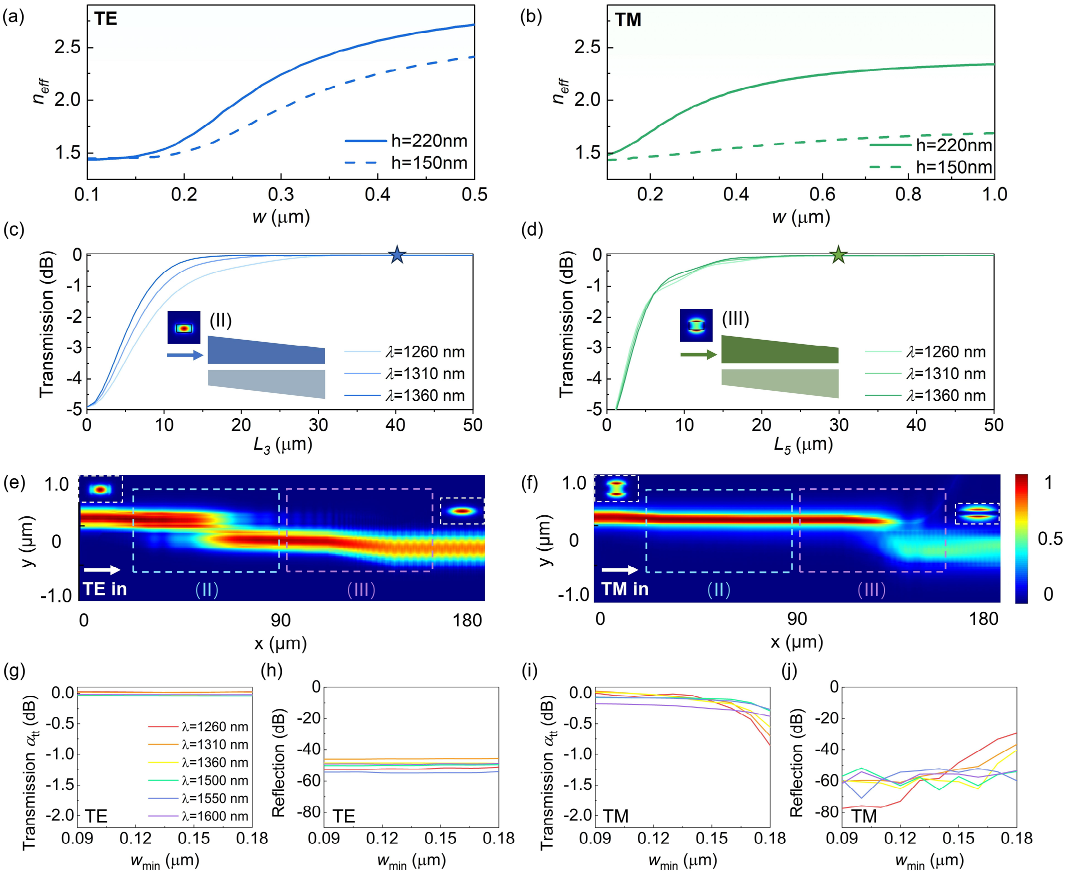

Figure S2: Design and analysis of the mode transition region. (a)-(b): Calculated effective index for waveguides A and B as the core width varies when operating at the wavelength of 1310 nm. (c)-(d): Calculated transmission at different wavelength in section II/III for TE/TM polarization mode coupling as the length L_3/L_5 varies. (e)-(f): Simulated light propagation of the entire mode transition region. (g)-(j): Calculated transmission loss α_{tt} and reflection as w_{min} varies.

S3. Simulation of the conventional bi-level inverse taper structure

Figure S3 shows the illustration of the EC based on a conventional bi-level inverse taper [1], which includes a mode transition region and a mode expanding region. Here the fundamental modes are evolved from the thick core to the thin core, where an inevitable bi-level junction is introduced at the interface. In this case, the critical feature size w_{min} refers to the taper tip width of the upper layer, playing a crucial role in the performance of the EC.

The mode conversion loss α_{tt} and the reflection of the mode transition region using the bi-level inverse taper structure is calculated as a comparison, as shown in Figure S4. The critical feature size w_{min} considered varies from 40 nm to 180 nm, covering most of the current mainstream fabrication node processes. The length of the bi-level taper is set as 30 μm, which is sufficiently long for the mode conversion under different w_{min} (see Figure S5). The bandwidth is also extended to 1600 nm for a more comprehensive analysis. The core width w of the input singlemode waveguide is chosen as 0.38 μm and 0.45 μm, a value that commonly used at the O- and C-band, respectively.

For the TE polarization mode, the bi-level inverse taper structure experiences low transmission loss of <0.048 dB across a broad wavelength range of 1260-1600 nm. However, the reflection is about –32.2-–48.3 dB when w_{min} varies from 40 nm to 180 nm, which is around 10 dB higher than that observed in our proposed coupling scheme (<–57.1 dB) with an asymmetric bi-level dual-core mode converter.

When it comes to the TM polarization mode, the mode conversion loss α_{tt} and reflection loss suffer from an evident deterioration as w_{min} varies and one can see that it is more sensitive to the width deviation. It has a transmission loss of 0.33-0.74 dB for the O-band operation when w_{min} varies from 90 nm to 130 nm. When w_{min} is increased to 160 nm, the conventional bi-level inverse taper has a transmission loss as high as 0.83-0.91 dB. It is noteworthy that even when considering an ultra-small feature size of 40 nm, the transmission loss is approximately 0.08-0.17 dB.

When the bandwidth is further extended to 1600 nm, an increased transmission loss of 1.02 dB is observed, while the calculated reflection is as high as −22.4-−47.7 dB in the wavelength range of 1260-1600 nm. It is worth noting that there is no significant difference when the width of the input singlemode waveguide w is chosen as 0.38 μm or 0.45 μm.

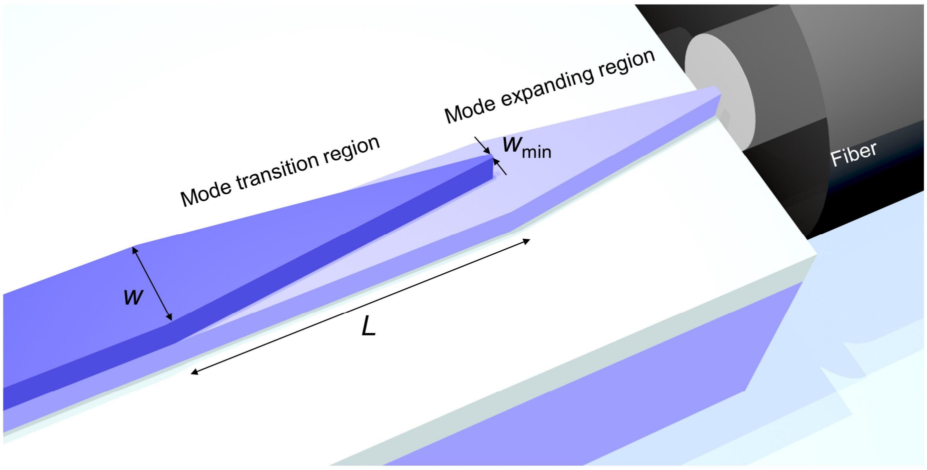

Figure S3: Illustration of the EC based on conventional bi-level inverse taper.

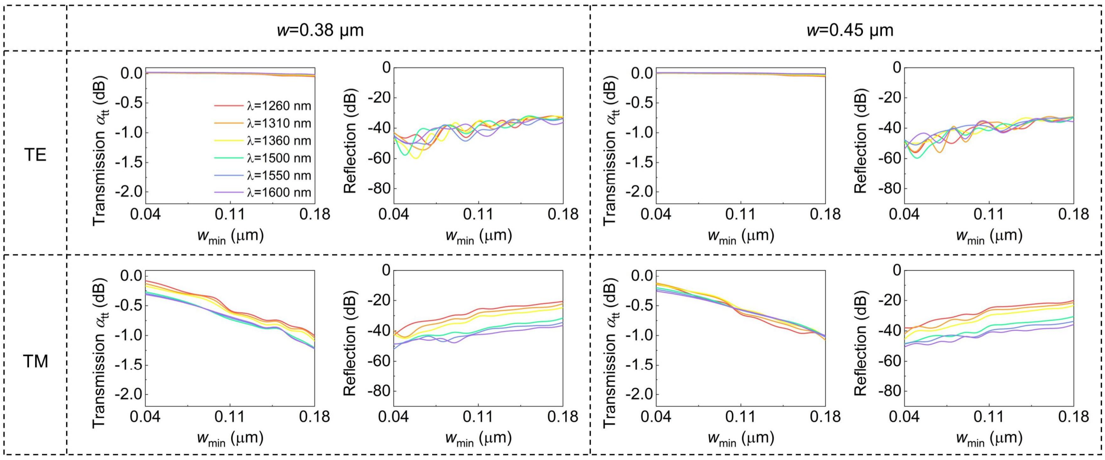

Figure S4: Calculated transmission loss α_{tt} and reflection of the mode transition region as w_{min} varies when w is chosen as 0.38 μm and 0.45 μm for TE/TM polarization modes.

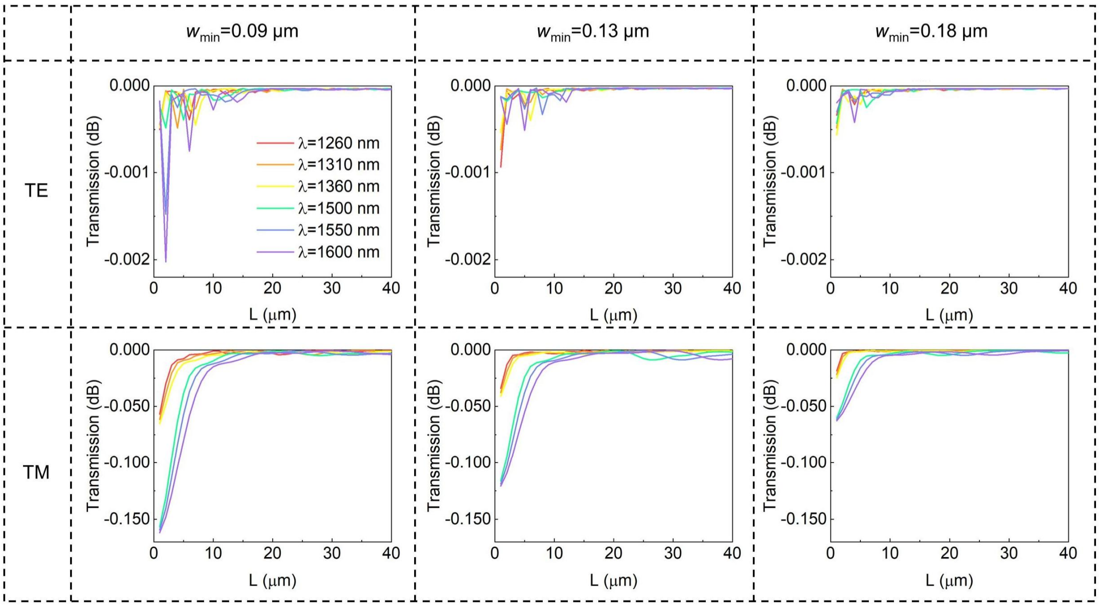

Figure S5: Simulated transmission of the bi-level inverse taper structure as the taper length L varies under different minimum feature size w_{min}.

S4. Fabrication tolerance analysis of the mode transition region

The fabrication tolerance analysis of the mode transition region is also carried out assuming that the core height or width has a deviation of ±10 nm and ±20 nm. Here we consider the case where the core width deviation is Δw, while assuming that the center-to-center separation between the two cores remains unchanged, and consequently the gap between the two cores exhibits a deviation of -Δw. As shown in Figure S6 (a) and (b), for the TE polarization mode, the on-chip transmission loss is <0.04 dB across the O-band when Δh=±20 nm. The maximum transmission loss of 0.17 dB occurs at the short wavelength when Δw=+20 nm. As shown in Figure S6 (c) and (d), for the TM polarization mode, an increased loss of ~0.12 dB is observed at the wavelength of 1260 nm when Δh=+20 nm, which is possibly due to the insufficient evanescent coupling. The transmission loss is <0.25 dB when Δw=±20 nm.

Furthermore, we have also given an analysis by assuming that the gap width individually has some deviation of Δw_g, while the core width remains unchanged. As shown in Figure S6 (e) and (f), it has an

additional loss of < 0.08 dB even when the gap deviation is as large as ±20 nm for both polarizations.

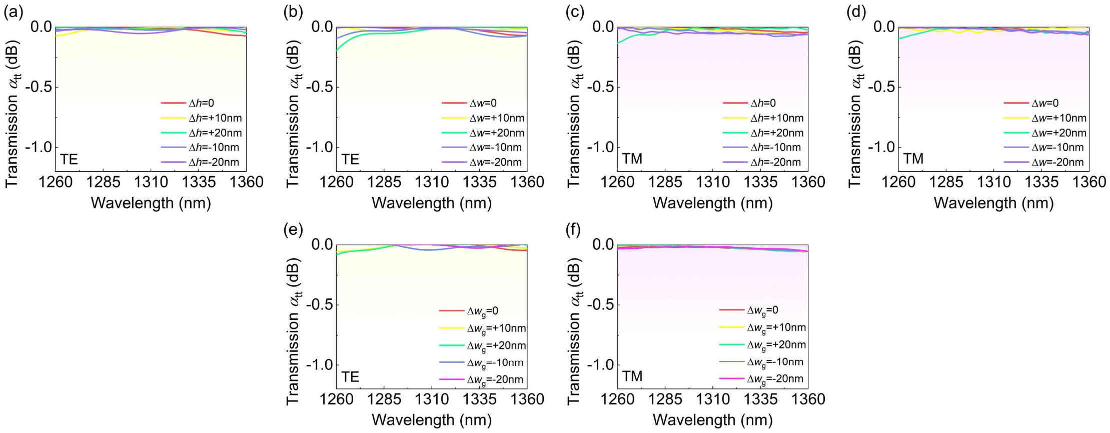

Figure S6: (a)-(d) Simulated transmission loss α_{tt} when assuming the core height or width has a deviation of ±10 nm and ±20 nm. (e)-(f): Simulated transmission loss α_{tt} by assuming that the gap width individually has a deviation of ±10 nm and ±20 nm, while the core width remains unchanged.

S5. Simulation of the mode expanding region

The mode expanding region (section IV) is designed by being divided into several inverse-taper sections so that the footprint can be minimized. All the taper lengths (L_6, L_7, L_8, L_9) for these sections are carefully chosen to minimize the on-chip transmission loss α_{te}. Finally, the widths (w_{b6}, w_{b7}, w_{b8}) and the lengths (L_6, L_7, L_8) are optimally chosen as (0.80, 0.50, 0.25) μm and (40, 10, 30) μm to ensure the adiabaticity. Here the gap w_{g3} at the end is chosen as 2 μm. For the section at the facet, there is a trade-off between the mode conversion loss and the substrate leakage loss as the mode field is mainly expanded to be beyond the confinement of the core region, and thus the length L_9 should be determined carefully. Figure S7(a) and (b) show the calculated transmission loss α_{te} and the substrate leakage loss as the length L_9 varies. It is important to mention that the thickness of the buried oxide layer, h_{BOX}, may influence the substrate leakage loss crucially. Additional analyses are also carried out with different buried oxide layer thicknesses h_{BOX}. As can be seen, the TE polarization mode suffers less substrate leakage than the TM polarization mode. When h_{BOX} is chosen as 2 μm, there is notable substrate leakage as the length L_9 increases, thereby increasing the transmission loss. On the other hand, however, the mode conversion might become non-adiabatic if the length is not sufficiently long. In this case, the transmission efficiency is also limited. Alternatively, it is possible to significantly reduce the substrate leakage and achieve a low transmission loss when choosing h_{BOX} =3 μm. In this case, the calculation result shows that the substrate leakage loss is reduced to 0.08-0.24 dB when L_9 is chosen as 50 μm, while the transmission loss α_{te} is as low as 0.26/0.27 dB for TE/TM polarization mode. Note that the substrate leakage still increases slightly, especially at longer wavelengths, as the length L_9 increases. Even though increasing the BOX thickness further to e.g. 4 μm helps reduce the substrate leakage, we have considered a design with h_{BOX} = 3 μm, which is a commonly available commercial value.

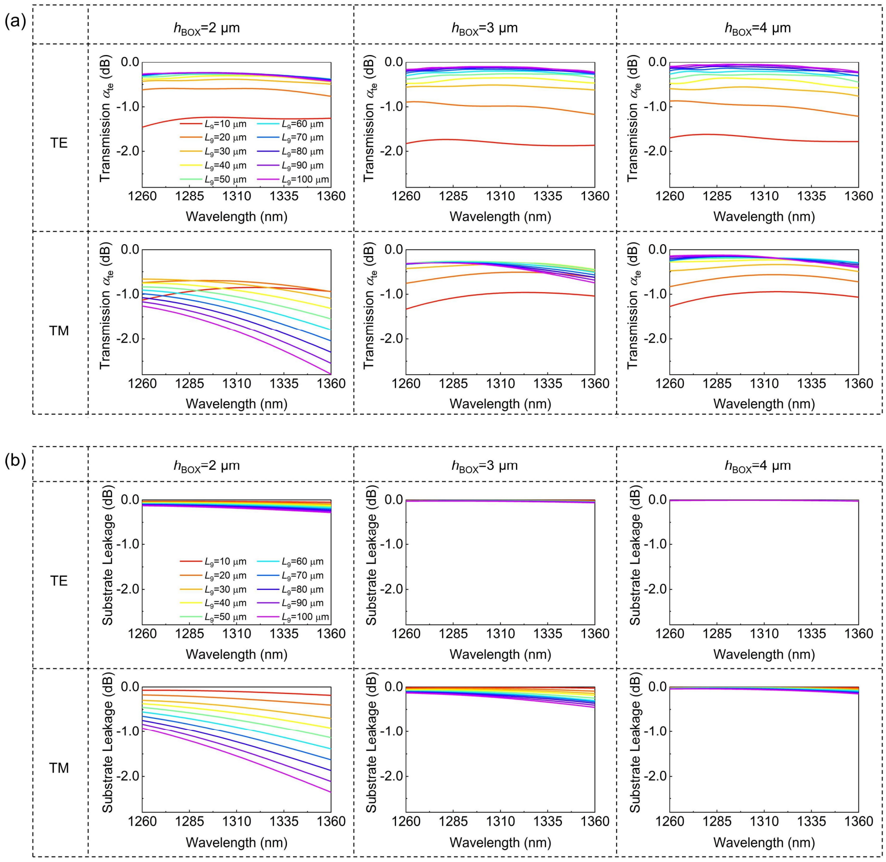

Figure S7: Design and analysis of the mode expanding region. Calculated transmission loss α_{te} (a) and the substrate leakage loss (b) of the last section at the facet as the length L_9 varies when considering different cases of buried oxide layer thickness h_{BOX}.

S6. Analysis of the air gap between fiber and chip

We conduct a comprehensive analysis of the influence of the air gap between the optical fiber and the silicon photonic chip with the proposed EC, the calculated coupling loss are depicted in Figure S8. When the gap is about 0.5 μm, a noticeable loss deterioration ranging from 0.35 dB to 0.43 dB and 0.31 dB to 0.40 dB is observed for TE and TM polarization mode, respectively. We then gradually increased the gap to 3 μm, and the corresponding additional loss is about 1.10-1.20 dB and 1.40-1.59 dB, which also results in the reflection at the chip facet and results in ripples in the measured spectra.

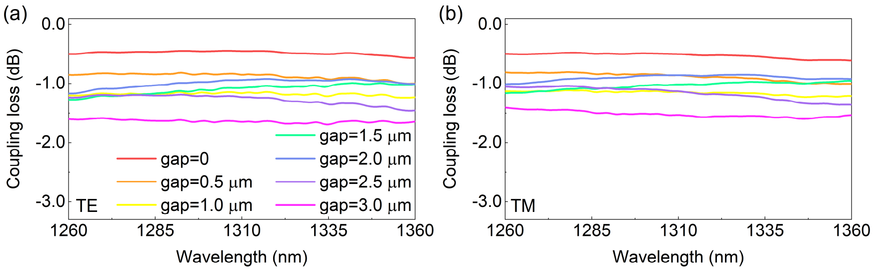

Figure S8: Calculated coupling loss when consider there is an air gap between fiber and chip. (a) TE, (b) TM polarization mode.

S7. Broadband simulation

We carried out the broadband simulation of the overall coupling loss under different w_{min} by extending the bandwidth range to 1260-1600 nm, as shown in Figure S9. The convergence width is 130 nm, i.e., the transmission loss for 130 nm and 90 nm is comparable. When the critical dimension is 90 nm or 130

nm, the 1-dB bandwidth of TE/TM polarization mode reaches 340/230 nm, respectively. Note that the TM polarization mode experience loss degeneration at the short wavelength as the critical dimension increases since the mode confinement is stronger and thus results in larger mode mismatch loss. The larger coupling loss for the TM polarization mode at the longer wavelength mainly comes from the non-negligible substrate leakage loss as well as the mode mismatch loss between the chip mode and the fiber mode. In order to analysis the loss composition more intuitively, we calculate the loss of each section at 1500-1600 nm, including the mode mismatch loss $\alpha_{c,}$ the transmission loss α_{tt} of the mode transition region and α_{te} of the mode expanding region, as shown in Figure S10. It can be seen that when operating at the longer wavelength, the increased coupling loss mainly attributes to the considerable substrate leakage loss and mode conversion loss α_{te} (~0.94-1.86 dB) in the mode expanding region, because the optimized waveguide parameters for O-band exhibits less mode confinement than at the C-band, especially for the TM polarization modes with vertical field distribution. This problem can be solved by introducing a thicker buffer layer or choosing a thicker waveguide, as analyzed in Section 2.3 and Supplementary S5. Furthermore, a smaller critical size is not required at the longer wavelength, which will conversely increase the transmission loss.

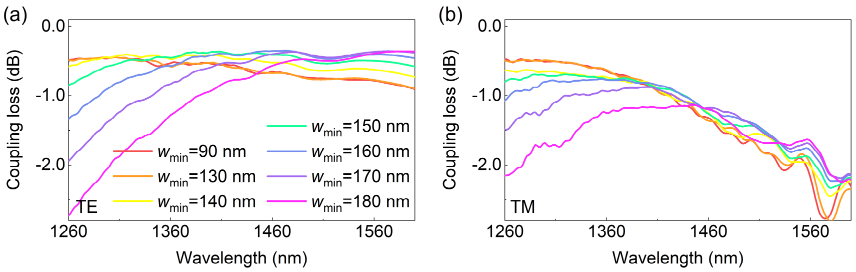

Figure S9: Simulated overall coupling loss in the wavelength range of 1260-1600 nm. (a) TE, (b) TM polarization mode.

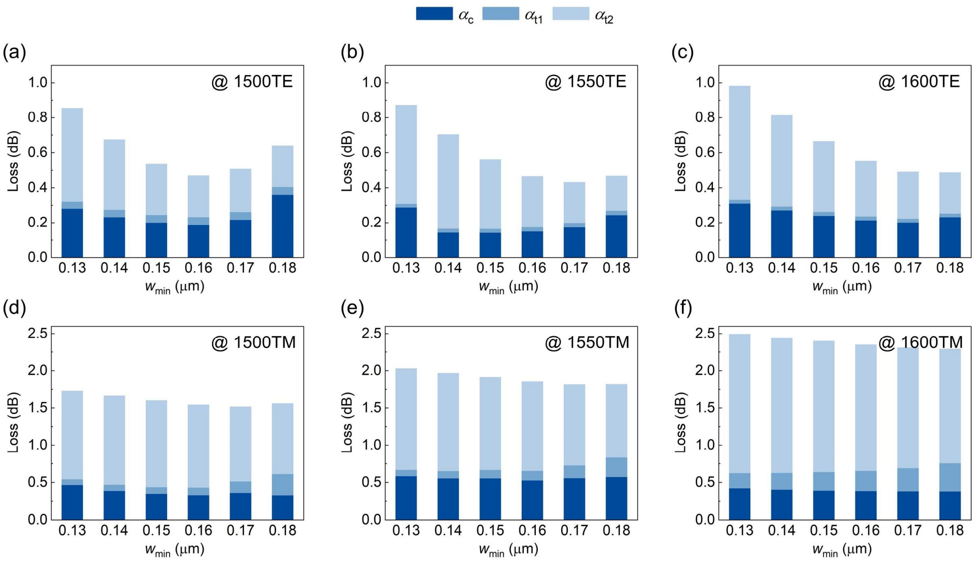

Figure S10: Calculated mode mismatch loss $\alpha_{c,}$ the transmission loss α_{tt} of the mode transition region and loss α_{te} of the mode expanding region as the tip width varies when operating at the wavelength of 1500 nm, 1550 nm and 1600 nm.

Reference

[1] L. Jia, C. Li, T.-Y. Liow, and G.-Q. Lo, "Efficient Suspended Coupler With Loss Less Than −1.4 dB Between Si-Photonic Waveguide and Cleaved Single Mode Fiber," *Journal of Lightwave Technology*, vol. 36, no. 2 pp. 239–244, 2018, https://doi.org/10.1109/JLT.2017.2779863.
